# Shexiang Baoxin Pills as an Adjuvant Treatment for Chronic Heart Failure: A System Review and Meta-Analysis

**DOI:** 10.1155/2018/6949348

**Published:** 2018-04-24

**Authors:** Taiwei Dong, Jian Wang, Xiao Ma, Rong Ma, Jianxia Wen, Nian Chen, Qian Xie

**Affiliations:** School of Pharmacy, Ministry of Education, Key Laboratory of Standardization of Chinese Medicine, Chengdu University of Traditional Chinese Medicine, Chengdu 611137, China

## Abstract

**Background:**

Shexiang Baoxin pills (SXBXP), as a Traditional Chinese Medicine, are widely used for chronic heart failure in China. It is essential to systematically assess the efficacy and safety of SXBXP as an adjuvant treatment for chronic heart failure.

**Methods:**

Seven English and Chinese electronic databases (PubMed, Embase, Cochrane Library, CBM, Wanfang, VMIS, and CNKI) were searched from inception to July 2017. The Cochrane Risk of Bias tool was used to evaluate the methodological quality of eligible studies. Meta-analysis was performed by Review Manager 5.3.

**Results:**

A total of 27 RCTs with 2637 participants were included in this review. Compared to conventional treatment, SXBXP combined with conventional treatment showed potent efficacy when it came to the total efficacy rate (OR, 3.88; 95% CI, 2.87, 5.26; *P* < 0.00001), B-type natriuretic peptide (BNP) (MD = −66.95; 95% CI, −108.57, −25.34; *P* = 0.002), N-terminal pro-brain natriuretic peptide (NT-ProBNP) (MD = −0.15; 95% CI, −0.21, −0.09; *P* < 0.00001), six-minute walking distance (6-MWD) (MD = 38.57; 95% CI, 28.47, 48.67; *P* < 0.00001), cardiac output (CO) (MD = 0.84; 95% CI, 0.68, 0.99; *P* < 0.00001), and Stroke Volume (SV) (MD = 7.43; 95% CI, 4.42, 10.44, *P* < 0.00001). The pooled subgroup analysis indicated that there was a significant difference between SXBXP plus conventional treatment and conventional treatment alone in short term course (OR = 3.51; 95% CI, 2.28, 5.40; *P* < 0.00001), in middle period of treatment (OR = 5.01; 95% CI, 2.61, 9.60; *P* < 0.00001), and in long-term course (OR = 3.77; 95% CI, 2.13, 6.67; *P* < 0.00001). No serious adverse events or reactions were mentioned in these RCTs.

**Conclusions:**

As an adjuvant drug, this study suggested that SXBXP provide an obvious efficacy for the treatment of CHF. However, due to small samples and generally low quality studies being applied in this study, more rigorous and well-designed RCTs are needed to confirm these findings.

## 1. Introduction

In spite of a tremendous advance in pharmacology and therapies, chronic heart failure (CHF) remains the most serious cardiovascular disorder all over the world [1, 2]. There is a significant high mortality in patients with CHF [3], probably an estimated 50% mortality in 5 years [4]. Moreover, with the aging of the population becoming a more and more serious issue, patients with CHF constitute a high proportion of the aging population, and so patients with CHF increase year after year, which highlights the urgent need for effective treatment strategies [5].

CHF is a complex clinical syndrome that results from structural change or functional abnormalities, which may lead to a series of cardiac dysfunctions, such as decrease of cardiac output, increase of intracardiac pressure function, ventricular filling, or impaired ejection at both rest and load conditions [6]. Cardinal manifestations are various, such as dyspnea and fatigue, which may lead to fluid retention due to limited exercise tolerance and which may bring about pulmonary and peripheral edema [7]. CHF is the terminal stage of various heart diseases, often accompanied by high morbidity and high mortality, especially in the elderly [8]. Patients with CHF died within 5 years as well as the survival rate being less than 50% within 1 year [9]. CHF poses a serious challenge to the health of the people of the world, giving the family and society a heavy burden.

Current Western medicine treatment formed with “angiotensin-converting enzyme inhibitor (ACEI) or Angiotensin receptor antagonist (ARB), beta blockers, or Aldosterone receptor antagonist” is the basis of the Golden Triangle treatment program [2]. However, satisfactory results are still difficult to obtain in some patients. A lot of researches show that, in CHF patients with Yang deficiency blood stasis, SXBXP have beneficial Qi Tong Yang Huayu efficacy and played a very effective role in the treatment of CHF. These Western medicine treatments are not only conventional treatments, but also the dominating treatment. However, the existing treatment is not perfect enough [10]. It is well known that long-term use of Western medicine may cause side effects and resistance [11].

SXBXP, as a traditional and complementary medicine, derives from traditional decoction named Suhexiang pills, which is recorded in the Ministry of Health and the benefits of the party side in Song Dynasty. The traditional decoction has been used for more than a thousand years and it includes* Moschus*,* toad*,* Panax ginseng*,* Bos taurus domesticus Gmelin, Cinnamomum cassia Presl,* and* Borneolum *[12]. From 1981, since the clinical application, SXBXP was widely used in coronary heart disease, angina, myocardial infarction, and other heart diseases. Most clinical trials showed that SXBXP benefited patients with CHF [13]. Now, a large number of references reported the efficacy of SXBXP on CHF. All the trails did not report obvious side effects and adverse reactions. However, evidence was very limited on the efficacy of SXBXP for CHF [14]. In fact, the previous systematic review did not assess the effects of SXBXP as an adjuvant treatment for CHF, too. Therefore, it is necessary for us to assess the efficacy and safety of SXBXP, which act as an adjuvant treatment with conventional treatment.

## 2. Materials and Methods

### 2.1. Search Strategy

Comprehensive searches were conducted in both English and Chinese databases to identify all published RCTs from their inception to July 2017. All relevant RCTs were searched from the following 7 databases including PubMed, Embase, Cochrane Library, CBM, Wanfang, VMIS, and CNKI. The following search terms were used: “Shexiang Baoxin pills” [Title/Abstract] AND “Chronic heart failure” [Title/Abstract] OR “Chronic heart disease” [Title/Abstract]. The literature searches were independently examined by two investigators (Taiwei Dong and Rong Ma) and disagreements were resolved by consensus as well as discussion. The bibliographies of included trials were searched for through references. However, the trials without English abstract would be translated by the investigator Taiwei Dong and checked by the investigator Rong Ma.

### 2.2. Inclusion Criteria

Two authors (Taiwei Dong and Rong Ma) read the titles and abstracts of trials in all searched databases independently to assess the rationality for inclusion. The full text was further read to evaluate for the inclusion criteria. The inclusion criteria were as follows. (1) Investigative object and intervention: all the randomized controlled trails (RCTs) which combined SXBXP with conventional medical treatment (experimental group) compared with conventional medical treatment (control group) alone in CHF were included. The method of intervention was oral administration. (2) Characteristics of patients: patients diagnosed with CHF with New York Heart Association (NYHA) (The Criteria Committee of the New York Heart Association 1994) classified from II to IV were included. (3) Outcome measures: total efficacy rate. The secondary outcome measures included left ventricular ejection fraction (LVEF), cardiac output (CO), Stroke Volume (SV), B-type natriuretic peptide (BNP), N-terminal pro-brain natriuretic peptide (NT-ProBNP), and six-minute walking distance (6-MWD). RCTs with one or more outcomes were included.

### 2.3. Exclusion Criteria

The trials conforming to the following conditions were excluded: (1) reduplicative publications reporting the same trials; (2) nonrandomized controlled trials; (3) nonclinical experiments, reviews, literature research, mechanism research, or animal experiment; (4) controlled interventions combined with any other Chinese herbal medicine or acupuncture in control group or experimental group; (5) unavailable or incorrect data for meta-analysis; (6) patients with unclear functional classification; (7) trials with unclear evaluation indicators or basic data for statistic research.

### 2.4. Data Extraction

Two investigators (Taiwei Dong and Rong Ma) independently extracted the basic information such as title, published year, total cases, cases of experiment group and control group, interventions, outcome measures, NYHA classification, course of disease, safety evaluation, and ADEs or ADRs to conclusive tables. Relevant disagreements were resolved through discussion with investigator (Jian Wang). Symptom improvement was evaluated according to the Guidance for Clinical Research on New Drugs of TCM, Framingham criteria, American College of Cardiology/American Heart Association (ACC/AHA), or textbook criteria as long as the criteria met the international-used diagnostic criteria [15].

### 2.5. Risk of Bias Assessment and Quality Assessment

The methodological quality of included RCTs was assessed by Review Manager 5.3 according to the Cochrane Risk of Bias tool. The methodological quality of each trial was evaluated by seven domains including random sequence generation (selection bias), allocation concealment (selection bias), blinding of participants and personnel (performance bias), blinding of outcome assessment (detection bias), incomplete outcome data (attrition bias), selective reporting (reporting bias), and other biases. The quality of each trial was classified as “high risk,” “unclear risk,” or “low risk.” The trials that had insufficient information available to make a judgment were classified as unclear risk of bias. The trials with low risk of bias represented a good methodological quality and the trials with high risk of bias represented a low methodological quality. Any disagreement was settled through discussion with investigator (Jian Wang).

### 2.6. Strategy for Data Synthesis and Analysis

The meta-analysis was performed by Review Manager 5.3 software (Cochrane Collaboration, Oxford, UK). For outcome measures, dichotomous variables were presented as odds ratio (OR) with 95% confidence intervals (CI), while continuous outcomes were expressed as mean difference (MD) with 95% CI. As a quantitative measure of inconsistency, the *I*-square (*I*^2^) statistic was used to assess heterogeneity. Fixed effect model was performed with minor heterogeneity when *I*^2^ was less than 50%. Random-effect model was applied when *I*^2^ was over 50%. A funnel plot was used for assessing the potential publication bias. Furthermore, subgroup analysis was performed due to course of treatment of SXBXP.

## 3. Results

### 3.1. Basic Information

#### 3.1.1. Description of Studies

**Figure 1:**
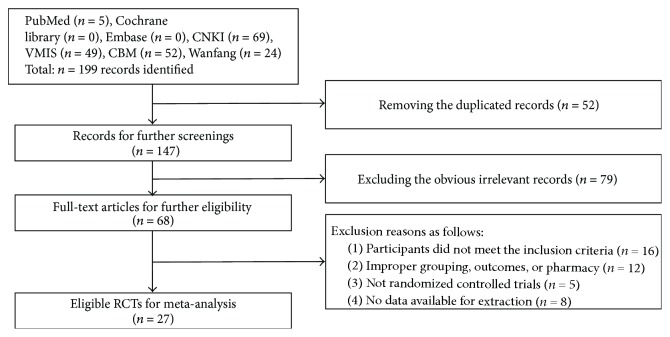
Flow diagram for searching and selecting study.

A total of 199 records were identified for preliminary screening after searching English and Chinese databases. All the included trials were conducted in China and published in Chinese. As shown in Figure 1, 147 records were reserved for further screening after removing 52 duplicated publications. For the preserved records, 79 obvious irrelevant literatures were excluded by reading the title and abstract. 68 full-text articles were used for further assessment. After reading the full text, 41 more literatures were excluded for the following reasons: participants not meeting the inclusion criteria (*n* = 16), improper grouping, outcomes, or pharmacy (*n* = 12), nonrandomized controlled trials (*n* = 5), and no data available for extraction (*n* = 8). Finally, 27 RCTs of SXBXP for CHF were included in this review.

#### 3.1.2. Study Characteristics

As shown in Table 1, a total of 27 RCTs with 2637 participants were included in this review. The control group consisted of 1313 patients, while the treatment group consisted of 1324 patients. All trials' sample sizes ranged from 56 to 181; sample size is large enough. The ages of the subjects were over 50 years. Moreover, all the trials included NYHA classification among II~IV. As for the characteristics of intervention, the course of treatment varied from 24 days to 6 months. Only one trial did not mention the course of treatment [16]. The baseline of patients in both groups was balanced.

The treatment group used SXBXP combined with the same conventional treatment as control group; only one trial used Placebo combined with conventional treatment [17]. Two groups of all trails used the dose of 202.5 mg/d, although most doses are 135 mg/d; only one trial used 67.5 mg/d. SXBXP was given through oral administration three times daily in all included trials. The control group used conventional medical treatment alone, including ACEI, ARB, cardiac glycosides, diuretics, *β*-receptor blockers, antialdosterone drugs, calcium channel blockers, or vasodilators.

None of the included trials reported death. All trails reported NYHA classification. Twenty-two trials reported LVEF. Eighteen trials reported ER and 6 MWT. Four articles reported NT-proBNP. Four articles reported BNP. Eight trails reported CO. Seven trails reported SV.

### 3.2. Methodological Quality

**Figure 2:**
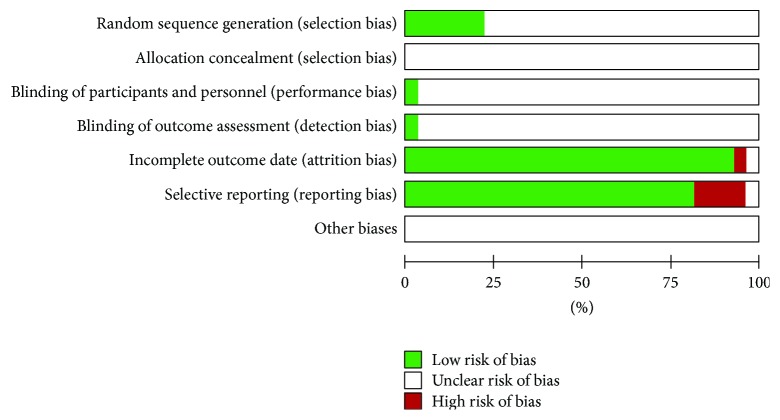
Risk of bias assessment of included studies.

The methodological quality of the included trials was generally poor. Five trials reported that random sequence was generated by a random number table [18] and only one trail generated it by the envelope method [19]; the remaining 21 trials only mentioned random allocation without any description of the method of randomization. There was no allocation concealment mentioned in all the articles. Only one trial mentioned blinding of participants and blinding of outcome assessment [17]. Two articles reported incomplete outcome data and selective reporting [20, 21]. About 20% trials showed high risk of bias in incomplete outcome data. Other potential sources of bias were unclear. Therefore, the quality of all the included trails was graded as high risk of bias. The details of the methodological quality were presented in Figure 2 and Table 2.

### 3.3. Publication Bias

**Figure 3:**
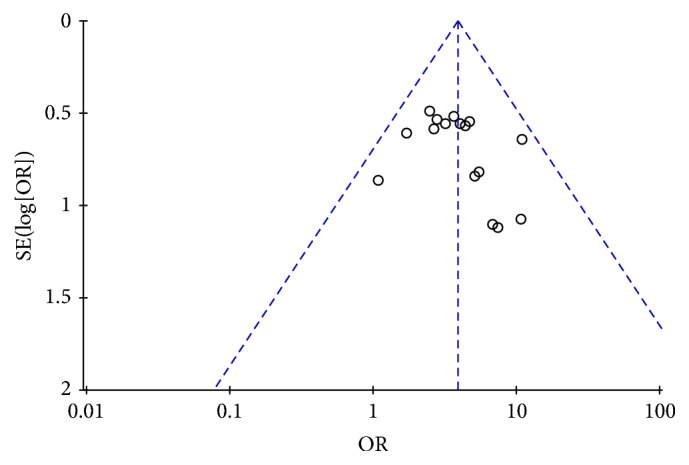
Funnel plot of SXBXP plus conventional treatment versus conventional treatment on total efficacy rate in patients with CHF.

Publication bias was assessed using a funnel plot based on the total efficacy rate reported in 18 trials. The funnel plot was asymmetrical, which indicated that the potential publication bias might influence the results of this review. The bias might result from these reasons: small sample size, poor quality, and a high proportion of positive results (Figure 3).

### 3.4. Effects of Interventions

#### 3.4.1. Primary Outcome Measures

**Figure 4:**
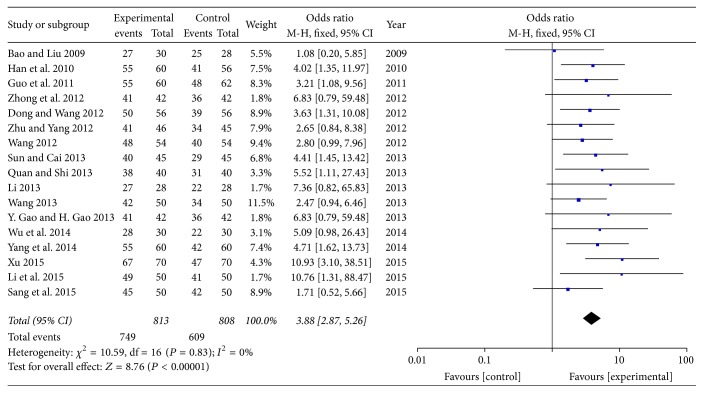
*Forest plot of SXBXP plus conventional treatment versus conventional treatment on total efficacy rate in patients with CHF*. *I*^2^ and *P* are the criteria for the heterogeneity test, ◆ is pooled mean difference; —■— is mean difference and 95% CI.

A total of 18 trials with 1735 patients investigated the total efficacy rate of SXBXP plus conventional treatment in improving NYHA classification in patients with chronic heart failure [18–20, 22–35]. There were 831 patients in experiment group and 904 in control group. The result showed that there was no heterogeneity (*P* = 0.83, *I*^2^ = 0%) and the fixed effect model was adopted for analysis. As shown in forest plot, there was a statistically significant difference between SXBXP plus conventional treatment and conventional treatment alone in the total efficacy rate (OR, 3.88; 95% CI, 2.87, 5.26; *P* < 0.00001) (Figure 4).

#### 3.4.2. Secondary Outcome Measures

**Figure 5:**
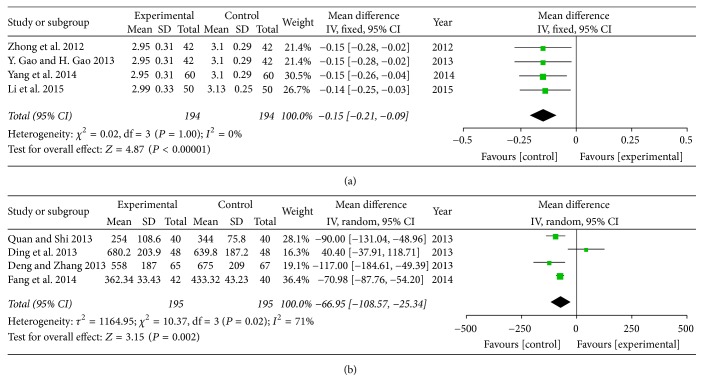
*Forest plot of SXBXP plus conventional treatment versus conventional treatment in the decrease of BNP and NT-Pro BNP*. *I*^2^ and *P* are the criteria for the heterogeneity test; ◆ is pooled mean difference; —■— is mean difference and 95% CI. (a) is forest plot of NT-Pro BNP; (b) is forest plot of BNP.

*(1) 1B-Type Natriuretic Peptide (BNP) and N-Terminal Pro-Brain Natriuretic Peptide (NT-Pro BNP). *Four trails with 390 patients assessed the therapeutic efficacy of NT-Pro BNP [17, 31, 36, 37]. Four trails with 388 participants assessed the effect of SXBXP plus conventional treatment in decreasing BNP in patients with chronic heart failure [27, 33–35]. It has considerably high heterogeneity in BNP (*P* = 0.02, *I*^2^ = 71%), and there is no significant difference between SXBXP plus conventional treatment and conventional treatment alone on NT-Pro BNP (*P* = 1.00, *I*^2^ = 0%). The trials reported that SXBXP plus conventional treatment was superior to conventional treatment alone to reduce NT-Pro BNP (MD = −0.15; 95% CI, −0.21, −0.09; *P* < 0.00001) (Figure 5(a)) and BNP (MD = −66.95; 95% CI, −108.57, −25.34; *P* = 0.002) (Figure 5(b)).

**Figure 6:**
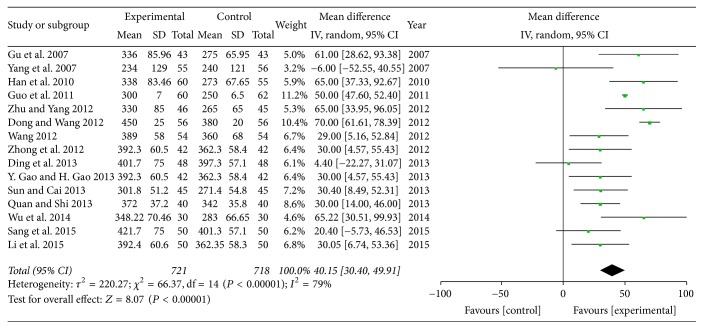
*Forest plot of SXBXP plus conventional treatment versus conventional treatment in increasing 6-MWD*. *I*^2^ and *P* are the criteria for the heterogeneity test; ◆ is pooled mean difference; —■— is mean difference and 95% CI.

*(2) The Comparison of 6-Minute Walking Distance (6-MWD). *A total of 15 studies with 1439 subjects reported the level of 6-MWD [17, 18, 24–31, 33, 34, 38, 39]. There was considerable heterogeneity (*P* < 0.00001, *I*^2^ = 79%) and random-effect model was conducted for analysis. The result showed that SXBXP could substantially increase the level of 6-MWD compared with conventional treatment (MD = 40.15; 95% CI, 30.40, 49.91; *P* < 0.00001) (Figure 6). It indicated a significant improvement of SXBXP for CHF in exercise endurance.

**Figure 7:**
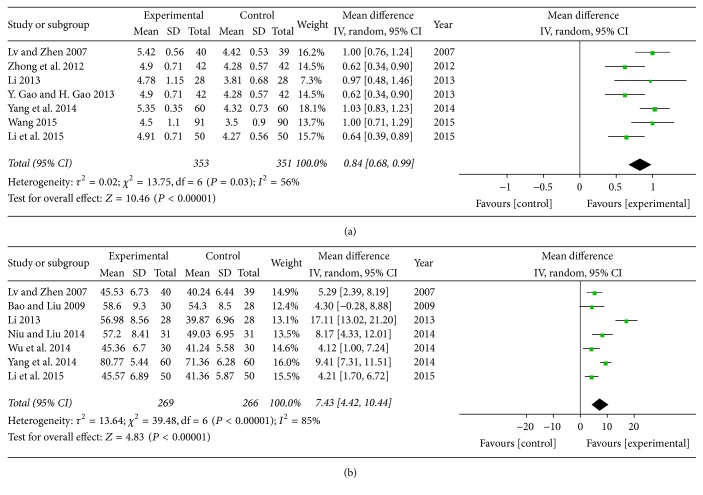
*Forest plot of SXBXP plus conventional treatment versus conventional treatment in increasing CO and SV*. *I*^2^ and *P* are the criteria for the heterogeneity test; ◆ is pooled mean difference; —■— is mean difference and 95% CI. (a) is forest plot of CO; (b) is forest plot of SV.

*(3) Cardiac Output (CO) and Stroke Volume (SV). *Seven trials with 704 participants [16, 17, 32–35, 40] and seven trials with 535 individuals [20, 23, 32, 34, 35, 40, 41] assessed the therapy on cardiac function of SXBXP plus conventional treatment in CO and SV, respectively. There was considerable heterogeneity in CO (*P* = 0.03, *I*^2^ = 56%) and SV (*P* < 0.00001, *I*^2^ = 85%) in trials. Meta-analysis with a random-effect model showed that, compared with conventional treatment, SXBXP plus conventional treatment significantly enhanced the cardiac function. The pooled analysis indicated that there was a statistically significant difference between SXBXP plus conventional treatment and conventional treatment alone on CO (MD = 0.84; 95% CI, 0.68, 0.99; *P* < 0.00001) (Figure 7(a)) and SV (MD = 7.43; 95% CI, 4.42, 10.44, *P* < 0.00001) (Figure 7(b)).

**Figure 8:**
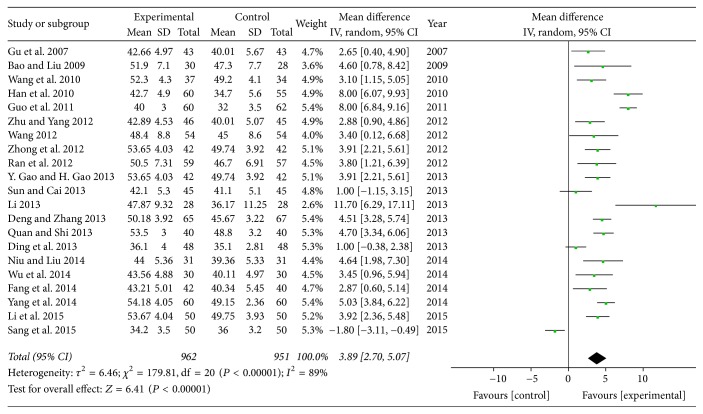
*Forest plot of SXBXP plus conventional treatment versus conventional treatment in increasing LVEF*. *I*^2^ and *P* are the criteria for the heterogeneity test; ◆ is pooled mean difference; —■— is mean difference and 95% CI.

*(4) Left Ventricular Ejection Fraction (LVEF). *A total of 21 trials evaluated LVEF and were pooled with a random model [17, 18, 20, 21, 23–27, 29–38, 41, 42]. The heterogeneity of the LVEF study was considerably high (*P* < 0.00001, *I*^2^ = 89%). Pooled comparisons demonstrated that SXBXP plus conventional treatment had a statistically significant beneficial effect compared to conventional treatment alone in terms of LVEF (MD = 3.89; 95% CI, 2.70, 5.07, *P* < 0.00001) (Figure 8). As an adjuvant drug, SXBXP improve the cardiac function of patients with CHF.

#### 3.4.3. Subgroup Analysis

**Figure 9:**
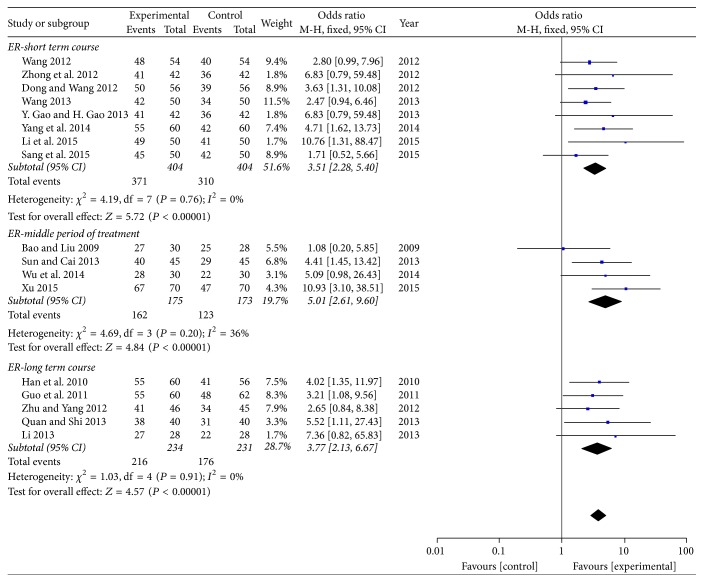
*Forest plot of SXBXP plus conventional treatment versus conventional treatment according to different courses in total efficacy rate*. *I*^2^ and *P* are the criteria for the heterogeneity test; ◆ is pooled mean difference; —■— is mean difference and 95% CI.

*(1) The Total Efficacy Rate with Different Courses. *Subgroup analysis was conducted to assess the efficacy of SXBXP plus conventional treatment on total efficacy rate according to different course of treatment. 8 trails with 681 participants were treated within 2 months [18, 19, 26, 28, 29, 33–35], which was regarded as short term course. The middle period of treatment included 4 trails with 285 patients; these participants were treated within 2–4 months [20, 22, 23, 30]. Moreover, the last subgroup contained 5 trails with 392 patients [24–26, 31, 32]; these participants were treated for over 4 months but less than 6 months. The *I*^2^ statistic of short term course showed that there was minor heterogeneity among these trials (*P* = 0.76, *I*^2^ = 0%). It also demonstrated similar result of the middle period of treatment (*P* = 0.20, *I*^2^ = 36%) and the long-term course (*P* = 0.91, *I*^2^ = 0%). All trails used fixed effect model to pool the results. The pool analysis presented that there was a significant difference between two therapy methods in short term course (OR = 3.51; 95% CI, 2.28, 5.40; *P* < 0.00001), in middle period of treatment (OR = 5.01; 95% CI, 2.61, 9.60; *P* < 0.00001), and in long-term course (OR = 3.77; 95% CI, 2.13, 6.67; *P* < 0.00001) (Figure 9).

## 4. Discussion

The basic pathogenesis of CHF is myocardial pathology “reconstruction.” CHF is the final stage of most heart diseases, but generally there are two processes that ultimately lead to CHF. One is the occurrence of myocardial death, such as myocardial damage caused by myocardial injury and severe myocarditis; the other is the neuroendocrine system caused by overactivation of systemic reactions [43, 44]. SXBXP can improve the heart pump function of CHF patients, reverse ventricular remodeling, and improve activity tolerance. The activation of the renin-angiotensin-aldosterone system (RAAS) and sympathetic nervous system leads to cardiac hypertrophy, which will result in ventricular remodeling and ultimately decompensation [45]. Therefore, delaying this process is particularly important for improving the quality of life and prolonging the survival time of patients with CHF. The indexes such as CO, SV, 6 MWT, LVEF, BNP, and NT-Pro BNP reflect the cardiac function and cardiac output. These indexes can be used to evaluate the cardiac function of patients with CHF and judge the CHF patients' clinical efficacy [46]. This study found that the combination of SXBXP and conventional treatment appeared to be more effective and safer for the treatment of CHF compared with conventional treatment. It indicated that SXBXP was worthy of clinical application and promotion.

SXBXP is a Chinese patent medicine. It is based on the work of Professor Ruihong Dai of Huashan Hospital, who belongs to Fudan University [47]. A group of Western medicine experts used the Western standard research and development of pure Chinese medicine preparations; it contains Moschus 6%, ginseng extract 27%, bezoar 4%, cinnamon 24%, storsin 16%, toad 4%, and* Dryobalanops* 19% [48]. Basic Research Certificate showed that storsin and* Dryobalanops* have slowed the heart rate and had the role of the lifting coronary artery spasm; Moschus extract can dilate blood vessels and enhance cardiac function effectively [49]. Ginseng saponins have antioxidant properties and positive muscle force and reduce the role of lipid [50]. Toad has a strong effect with heart [51].

This is the first systematic review to assess the effects of SXBXP as an adjuvant treatment for CHF. In this meta-analysis review, only two trails reported mild adverse reactions, and none of the included trails reported death. Thus, evidence is limited to make a summary of death and adverse reactions. So on the whole long-term use of SXBXP can be considered safe and effective. This result also proves several expert consensuses that the addition of Chinese medicines can improve the clinical symptoms and quality of life in patients with CHF, simultaneously maintain the cardiac function and reduce the rehospitalization rate, and depress mortality of patients with unique comment Western medicine treatment [52].

In this study, SXBXP displayed positive impact on clinical efficacy via increasing levels of LVEF and 6-MWT and reducing levels of NT-pro BNP and BNP. It seemed that SXBXP could ameliorate the cardiac function in patients with CHF according to primary outcomes, secondary outcomes, and subgroup analysis according to different course of treatment in experiment group.

The study implemented strict inclusion and exclusion criteria. However, there were still statistical heterogeneity between some of the outcome indicators of the included trials, due to the main consideration and the limited sample size and the variation in the length of treatment. Five issues still remain in all RCTs from the results: (1) the amount of included trials is small, in addition to the lack of high-quality and large sample study; (2) quality is generally low, random application is less, and blind implementation is unknown; (3) long-term follow-up is lacking; most studies did not mention mortality and readmission rate; (4) most of the studies did not report adverse reactions. Therefore, more high-quality with long-term follow-up RCTs were required to elucidate the effectiveness and security of SXBXP for CHF in the future.

## 5. Conclusions

In summary, as an adjuvant treatment for chronic heart failure, this study suggested that SXBXP have an obvious efficacy for the treatment of CHF. In the future, more multicenter, large sample size, randomized double-blind, long-term evaluation RCTs are needed to confirm the efficacy and mechanism of SXBXP for CHF.

## Figures and Tables

**Table 1:** Principal characteristics of the studies included in the meta-analysis.

Included research (year)	Sample size (*n*)		Age (y)		Male (%)		Intervening measure (T/C)	Dosage	Duration	Outcome measures
	T	C	T	C	T	C				
Gu et al. 2007	43	43	67.5 (8.8)	66.3 (8.2)	49%	53%	SXBXW + CT/CT	135 mg/d	6 M	LVEF, 6-MWD
Yang et al. 2014	60	60	65.9 (16.4)	64.3 (16.9)	72%	68%	SXBXW + CT/CT	135 mg/d	6 W	ER, LVEF, CO, SV, BNP
Sang et al. 2015	50	50	64.73 (5.03)	65.03 (5.39)	54%	44%	SXBXW + CT/CT	135 mg/d	24 D	ER, LVEF, 6-MWD
Ding et al. 2013	48	48	64.5 (7.2)	64.5 (7.2)	58%	58%	SXBXW + CT/Placebo + CT	135 mg/d	24 W	LVEF, 6-MWD, NT-Pro BNP
Deng and Zhang 2013	65	67	62 (11)	62 (11)	58%	58%	SXBXW + CT/CT	135 mg/d	12 W	LVEF, NT-Pro BNP
Zhong et al. 2012	42	42	52.4 (9.6)	53.1 (10.2)	52%	50%	SXBXW + CT/CT	135 mg/d	4 W	LVEF, 6-MWD, CO, NT-Pro BNP
Ran et al. 2012	59	57	61.5 (12.5)	64.3 (11.1)	46%	60%	SXBXW + CT/CT	135 mg/d	3 M	LVEF
Fang et al. 2014	42	40	64.35 (13.65)	65.98 (12.02)	43%	48%	SXBXW + CT/CT	135 mg/d	6 M	LVEF, NT-Pro BNP
Xu 2015	70	70	53.2 (7.3)	54.2 (6.2)	47%	46%	SXBXW + CT/CT	135 mg/d	3 M	ER
Wang 2015	91	90	48.5 (2.7)	48.7 (2.6)	57%	57%	SXBXW + CT/CT	202.5 mg/d	NR	ER, LVEF, CO
Bao and Liu 2009	30	28	65 (8.1)	65 (8.1)	66%	66%	SXBXW + CT/CT	135 mg/d	3 M	ER, LVEF, SV
Wang 2013	50	50	64.24 (4.95)	57.39 (6.21)	60%	64%	SXBXW + CT/CT	67.5 mg/d	28 D	ER
Yang et al. 2007	55	56	60 (8)	59 (9)	58%	54%	SXBXW + CT/CT	135 mg/d	6 M	6-MWD
Lv and Zhen 2007	40	39	65.2	67.1	58%	51%	SXBXW + CT/CT	135 mg/d	3 M	LVEF, CO, SV
Wu et al. 2014	30	30	64.2	65.7	57%	53%	SXBXW + CT/CT	135 mg/d	3 M	ER, LVEF, CO, SV
Li et al. 2015	50	50	61.6 (7.1)	61.8 (7.9)	52%	54%	SXBXW + CT/CT	135 mg/d	6 W	ER, LVEF, CO, SV, 6-MWD, NT-Pro BNP
Han et al. 2010	60	56	63.9 (16.4)	64.3 (16.9)	72%	73%	SXBXW + CT/CT	135 mg/d	6 M	ER, LVEF, 6-MWD
Niu and Liu 2014	31	31	73.5	76.5	61%	71%	SXBXW + CT/CT	135 mg/d	30 D	LVEF, SV
Sun and Cai 2013	45	45	58.5 (5.7)	59.2 (6.1)	67%	64%	SXBXW + CT/CT	135 mg/d	3 M	ER, LVEF, 6-MWD
Li 2013	28	28	68.5	69.5	64%	57%	SXBXW + CT/CT	135 mg/d	6 M	ER, LVEF, CO, SV
Wang 2012	54/	54	66	67	74%	72%	SXBXW + CT/CT	202.5 mg/d	1 M	ER, LVEF, 6-MWD
Y. Gao and H. Gao 2013	42	42	52.4 (9.6)	53.1 (10.2)	52%	50%	SXBXW + CT/CT	135 mg/d	4 W	ER, LVEF, CO, 6-MWD, NT-Pro BNP,
Wang et al. 2010	37	34	58.3 (5.4)	61.7 (6.5)	57%	56%	SXBXW + CT/CT	135 mg/d	4 M	LVEF, BNP
Zhu and Yang 2012	46	45	60.7 (7.3)	61.7 (8.1)	52%	51%	SXBXW + CT/CT	135 mg/d	6 M	LVEF, 6-MWD
Dong and Wang 2012	56	56	75.5 (1.2)	76.5 (1.4)	41%	38%	SXBXW + CT/CT	135 mg/d	4 W	ER, 6-MWD
Guo et al. 2011	60	62	63.9 (16.4)	64.3 (16.9)	55%	55%	SXBXW + CT/CT	135 mg/d	6 M	ER, LVEF, 6-MWD
Quan and Shi 2013	40	40	61	61	73%	73%	SXBXW + CT/CT	135 mg/d	6 M	ER, LVEF, 6-MWD, NT-Pro BNP

*Note*. NR: not reported; M: month; W: week; D: day; T: trail group; C: conventional group; CT: conventional treatment; SXBXP: Shexiang Baoxin pills.

**Table 2:** Risk of bias assessment of included studies.

Included research (year)	Random sequence generation	Allocation concealment	Blinding of participants and personnel	Blinding of outcome assessment	Incomplete outcome data	Selective reporting	Other biases
Gu et al. 2007	Unclear	Unclear	Unclear	Unclear	Low	Low	Unclear
Yang et al. 2014	Unclear	Unclear	Unclear	Unclear	Low	Low	Unclear
Sang et al. 2015	Low	Unclear	Unclear	Unclear	Low	Low	Unclear
Ding et al. 2013	Unclear	Unclear	Low	Low	Low	Low	Unclear
Deng and Zhang 2013	Unclear	Unclear	Unclear	Unclear	Low	Low	Unclear
Zhong et al. 2012	Unclear	Unclear	Unclear	Unclear	Unclear	Low	Unclear
Ran et al. 2012	Unclear	Unclear	Unclear	Unclear	Low	Unclear	Unclear
Fang et al. 2014	Low	Unclear	Unclear	Unclear	Low	Low	Unclear
Xu 2015	Low	Unclear	Unclear	Unclear	Low	Low	Unclear
Wang 2015	Unclear	Unclear	Unclear	Unclear	Low	Low	Unclear
Bao and Liu 2009	Unclear	Unclear	Unclear	Unclear	Low	Low	Unclear
Wang 2013	Low	Unclear	Unclear	Unclear	Low	Low	Unclear
Yang et al. 2007	Unclear	Unclear	Unclear	Unclear	Low	High	Unclear
Lv and Zhen 2007	Low	Unclear	Unclear	Unclear	High	Low	Unclear
Wu et al. 2014	Low	Unclear	Unclear	Unclear	Low	High	Unclear
Li et al. 2015	Unclear	Unclear	Unclear	Unclear	Low	Low	Unclear
Han et al. 2010	Unclear	Unclear	Unclear	Unclear	Low	Low	Unclear
Niu and Liu 2014	Unclear	Unclear	Unclear	Unclear	Low	Low	Unclear
Sun and Cai 2013	Unclear	Unclear	Unclear	Unclear	Low	Low	Unclear
Li 2013	Unclear	Unclear	Unclear	Unclear	Low	Low	Unclear
Wang 2012	Unclear	Unclear	Unclear	Unclear	Low	High	Unclear
Y. Gao and H. Gao 2013	Unclear	Unclear	Unclear	Unclear	Low	High	Unclear
Wang et al. 2010	Unclear	Unclear	Unclear	Unclear	Low	Low	Unclear
Zhu and Yang 2012	Unclear	Unclear	Unclear	Unclear	Low	Low	Unclear
Dong and Wang 2012	Unclear	Unclear	Unclear	Unclear	Low	Low	Unclear
Guo et al. 2011	Unclear	Unclear	Unclear	Unclear	Low	Low	Unclear
Quan and Shi 2013	Unclear	Unclear	Unclear	Unclear	Low	Low	Unclear

Unclear: unclear risk; Low: low risk; High: high risk.

## Abbreviations

SXBXP: Shexiang Baoxin pills
CHF: Chronic heart failure
NYHA: New York Heart Association
LVEF: Left ventricular ejection fraction
BNP: B-type natriuretic peptide
NT-ProBNP: N-terminal pro-brain natriuretic peptide
6-MWD: 6-minute walking distance
CO: Cardiac output
SV: Stroke Volume
ADEs: Adverse events
ADRs: Adverse drug reactions
ACEI: Angiotensin-converting enzyme inhibitors
ARB: Angiotensin receptor blocker
TCM: Traditional Chinese Medicine
OR: Odds ratio
CI: Confidence interval
MD: Mean difference.
